# Strategies and opportunities to control breast myopathies: An opinion paper

**DOI:** 10.3389/fphys.2023.1173564

**Published:** 2023-04-06

**Authors:** Richard A. Bailey

**Affiliations:** Aviagen Ltd, Newbridge, United Kingdom

**Keywords:** broiler, breast muscle, meat quality, myopathy, physiology

## Introduction

Chicken breast meat is one of the most sustainable and affordable sources of animal protein making it one of the most popular protein sources globally. As such, maintaining consistency in product quality is of the utmost importance. Over the last decade three novel myopathies have been identified in broilers (White striping (WS), Wooden breast (WB) and Spaghetti breast (SB)) (Soglia et al., 2019; Baldi et al., 2021; Barbut and Leishman, 2022); there has been a wealth of research across the poultry sector to understand their aetiology. These myopathies can be found together or individually in all broiler chicken breeds in all global regions (Lorenzi et al., 2014; Barbut, 2019; Soglia et al., 2019; Che et al., 2022a); the incidence and severity varies (Petracci et al., 2019; Che et al., 2022b) making them a complex area of study.

The exact aetiology of the myopathies is still not fully understood however a wide range of studies have used gene expression (Velleman and Clark, 2015; Zambonelli et al., 2016), proteomics (Kuttappan et al., 2017) and metabolomics (Boerboom et al., 2018) in an effort to characterise and understand the underlying biology. These studies have shown that muscle affected by the myopathies have an increased expression of genes associated with a range of metabolic, anatomical, and structural biological processes. Whilst the three myopathies are distinct from each other, the current understanding indicates a common root in hypoxia and oxidative stress resulting in disturbed growth and development within in the muscle (Petracci et al., 2019; Soglia et al., 2021). Whilst these studies indicate what is occurring in the affected muscle at the point of sampling it is still not clear what the initial triggers are.

## Current opportunities for control strategies

Genetic selection for broiler performance traits such as bodyweight (BW) and breast yield (BY) has been a core theory as a cause of the myopathies. Published data of large populations of broiler pure lines have estimated low genetic correlation between the three myopathies and performance traits (BY and BW), this indicates there is little shared genetic background between the myopathies and broiler performance traits (Bailey et al., 2015; 2020). Heritabilities for the myopathies were also estimated in these studies and found to be low to moderate (0.04 for SB, 0.024–0.097 for WB and 0.185–0.338 for WS). Alnahhas et al. (2016)reported a higher heritability for WS (0.65), where WS was measured on an underlying continuous scale rather than a categorical scale as per Bailey et al. (2015, 2020). According to Dempster and Lerner (1950) this can result in a higher heritability estimate; a heritability of 0.65 on the continuous scale would correspond to a lower heritability of 0.41 on the observed categorical scale. Another key difference is the fitting of the effect of the common maternal environment as done by Bailey et al. (2015, 2020). Alnahhas et al. (2016) did not fit this effect therefore this environmental effect is included in the genetic variance, thus over-estimating the heritability. The low to moderate heritabilities indicate that there is a genetic component to myopathy development but it is not the major contributing factor. Nevertheless, the genetic component can be used to select against the genetic propensity for developing the myopathies (Bailey et al., 2015). Empirical testing has shown that genetic selection for WB potentially reduces the relative incidence of WB by around 9.2% (Bailey et al., 2020), whilst at the same time continuing to improve breast yield through balanced breeding. Even though improvements can be made through genetic selection, these improvements are slow due to the low heritabilites of the myopathies and thus must be viewed as a long term strategy. The non-genetic factors should not be dismissed as they offer a more impactful and more immediate opportunity to reduce the incidence of myopathies as they have a significantly greater influence than the genetic factors.

There are many non-genetic factors that can influence broiler growth rates such as incubation, brooding, nutrition, temperature and ventilation (Bartov, 1987; Leksrisompong et al., 2009; Baracho et al., 2019). A key aspect of muscle growth and development are the satellite cells which drive growth and repair of muscle (Moss and Leblond, 1970), and play an important role in meat quality traits (Velleman, 2022). Incubation conditions influence early satellite cell development and can influence meat quality traits and may play a role in myopathy development (Oviedo-Rondón et al., 2020b; Halevy, 2020). During the first week post hatch these cells are most active and their population increases rapidly (Mann et al., 2011; Daughtry et al., 2017; Halevy, 2020). Satellite cell number and activity are negatively impacted upon if conditions during brooding are not optimal, e.g., elevated temperatures (Patael et al., 2019) or suboptimal early nutrition (Harthan et al., 2014; Powell et al., 2014; 2016; Velleman et al., 2014). It is therefore essential that the development of satellite cells is supported to maximize their potential to support optimal muscle development to reduce the risk of a myopathy occurring.

Oxidative stress and hypoxia have been highlighted as a key feature of all three myopathies (Soglia et al., 2021) therefore it is important to ensure optimal management though the whole life of the bird. Poor ventilation leading to poor oxygen availability or heat stress can lead to oxidative stress in the muscle increasing the risk of myopathies (Ain Baziz et al., 1996; Livingston et al., 2019a; Patael et al., 2019; Zaboli et al., 2019; Emami et al., 2021). With this in mind it is important to ensure that the environmental conditions within the broiler shed are in line with the breeder recommendations. Excessive build-up of carbon dioxide (>3000ppm, for example,) during brooding has been associated with an increased mortality and impaired cardiovascular function (McGovern et al., 2001; Olanrewaju et al., 2008) which will undoubtedly influence oxygen supply to the muscle.

Whilst genetic correlations indicate that there are no significant links between the myopathies and bird growth at the genetic level, phenotypically it is often the larger birds in a flock which express the myopathies. This phenotypic relationship is not always the case however, as some studies report that WS and WB are not linked to bird weight (Lorenzi et al., 2014; Trocino et al., 2015). Wooden breast and white striping do not occur spontaneously; chronological studies have shown that disruption to the breast meat at the cellular level can start as early as 2 weeks of age (Brothers et al., 2019; Chen et al., 2019) indicating that this could be a key time point to influence muscle development to reduce myopathy risk especially as growth rates start to increase from around 3 weeks of age (Aviagen, 2022). With this in mind, an important part of the strategy to control the myopathies could be to look at the growth trajectory of the birds and their breast muscles. Characterising the manner in which an individual bird reaches its final bodyweight and/or breast yield over time rather than the ultimate value may offer more insight into myopathy development and guide management strategies. Demand on the muscle for growth increases as the broiler reaches mid-phase growth; thus if there has been insufficient satellite cell development during early growth there may be an increased risk of myopathies occurring. In practical terms, any potential for accelerated growth later in life of the flock (e.g., following partial depopulation) could place increased demand on the muscle and pose a risk for myopathy development, particularly if early bird growth and satellite cell development was suboptimal.

One approach to influencing growth is reducing nutrient intake by diluting or limiting the availability of feed; these methods ultimately impact upon the efficiency of production through poor bird growth or the birds compensate by eating more food and thus do not offer a suitable solution (Meloche et al., 2018a; 2018b; Livingston et al., 2019b). By targeting specific amino acid levels or ratios the broiler growth curve can be influenced in a more elegant manner. Lysine is a key amino acid for muscle growth—when levels are reduced by 15% during mid-phase growth, WS and WB incidence is significantly reduced without impacting upon performance (Meloche et al., 2018c). Reduced incidence of WS and WB were also seen when the level of histidine (Lackner et al., 2022) or arginine (Zampiga et al., 2019) was increased relative to lysine. A theory for the success of altering the growth curve through mid-phase could be that it allows for the muscle support structures (e.g., the vascular system and connective tissues) to reach equilibrium with the muscle fibres prior to the next stage of growth.

Adoption of all-plant based diets has been considered by some to be a cause for the increase in myopathies due to reduced intake of dietary creatine which is found in diets containing animal by-products (Khan and Cowen, 1977; Ringel et al., 2007). Creatine supports muscle function by providing an alternative energy source to ATP/ADP (Wyss and Kaddurah-Daouk, 2000). Birds naturally produce creatine from arginine and glycine *via* the intermediate guanidinoacetic acid (GAA) (Portocarero and Braun, 2021) but this may divert these important amino acids away from other biologically important processes in the muscle such as blood vessel and connective tissue development (Oviedo-Rondón et al., 2020a). Exogenous GAA can be supplemented in the feed; when administered to broilers fed all-plant based diets it has been found to reduce the incidence of myopathies and increase breast meat yield (Córdova-Noboa et al., 2018a; 2018b).

Increasing dietary antioxidants such as vitamin E and selenium have been used to reduce oxidative stress and myopathies but results have been mixed and may depend upon the quality of fat in the diet (Guetchom et al., 2012; Kuttappan et al., 2012; 2021; Vieira et al., 2021). A novel approach to increase antioxidant levels was taken by increasing dietary phytase (Greene et al., 2019). Phytase breaks down phytate in the feed and releases inositol which is absorbed by the bird and taken up by the myoctyes. Greene et al. (2019), demonstrated that “super dosing” of phytase at a level of 2000 FTU significantly reduced the WB incidence and severity broilers, and also showed through metabolomics that it acted as an antioxidant with modulation of genes associated with oxygen homeostasis linking with a reduction in oxidative stress.

Spaghetti breast is probably the least understood myopathy (Baldi et al., 2021) and its incidence appears to be more sporadic than WB and WS making it more difficult to study. This myopathy is characterised by a loss of integrity of the muscle tissue which could indicate an insufficiency in the connective tissue in the muscle (Baldi et al., 2018; 2021; Soglia et al., 2021). Interestingly, in contrast to WB and WS, it is more likely to be found in female birds rather than males (Druyan et al., 2019; Pascual et al., 2020) which may offer avenues to understand its aetiology. In a study by Griffin et al. (2018), photographs of carcases of birds euthanised on farm were used to map the development of the three myopathies over time. Whilst WB and WS were easy to detect immediately *post mortem*, the authors stated that SB was not and thus not described fully due to the uncertainty; this raises the question of whether SB is present in the live bird (Petracci et al., 2019) or only detectable following *post mortem* change in the muscle. Immediately *post mortem*, muscle pH drops as a result of lactic acid production which is accompanied by the release of proteolytic enzymes (Etherington, 1984; Soglia et al., 2018; Lilburn et al., 2019). This process can ultimately soften connective tissue in the muscle (Etherington, 1984; Shi et al., 2021) and, in the event of an insufficiency in the connective tissue, could potentially cause SB to manifest. With that in mind it is possible that processes in the slaughter house could exacerbate the impact of *post mortem* changes in the muscle and thus increase SB incidence in a flock. The rate of cooling of carcases *post mortem* has an influence on the rate of lactic acid production and the activity of the proteolytic enzymes (Etherington, 1984; Mir et al., 2017; Shi et al., 2021)—the slower the rate of cooling the greater the opportunity for degradation of muscle (Huang et al., 2016). The use of compounds such as peracetic acid as part of meat hygiene measures may also impact connective tissue in a similar way to lactic acid so may play a role in the manifestation of SB. During plucking there is a manipulation of the carcases by the fingers on the pluckers — this physical interaction on the breast meat of the bird could disrupt the integrity of the connective tissue. As SB incidence remains highly variable these factors could offer areas to reduce incidence at the slaughter plant whilst the underlying aetiology is further investigated.

## Conclusion

**FIGURE 1:**
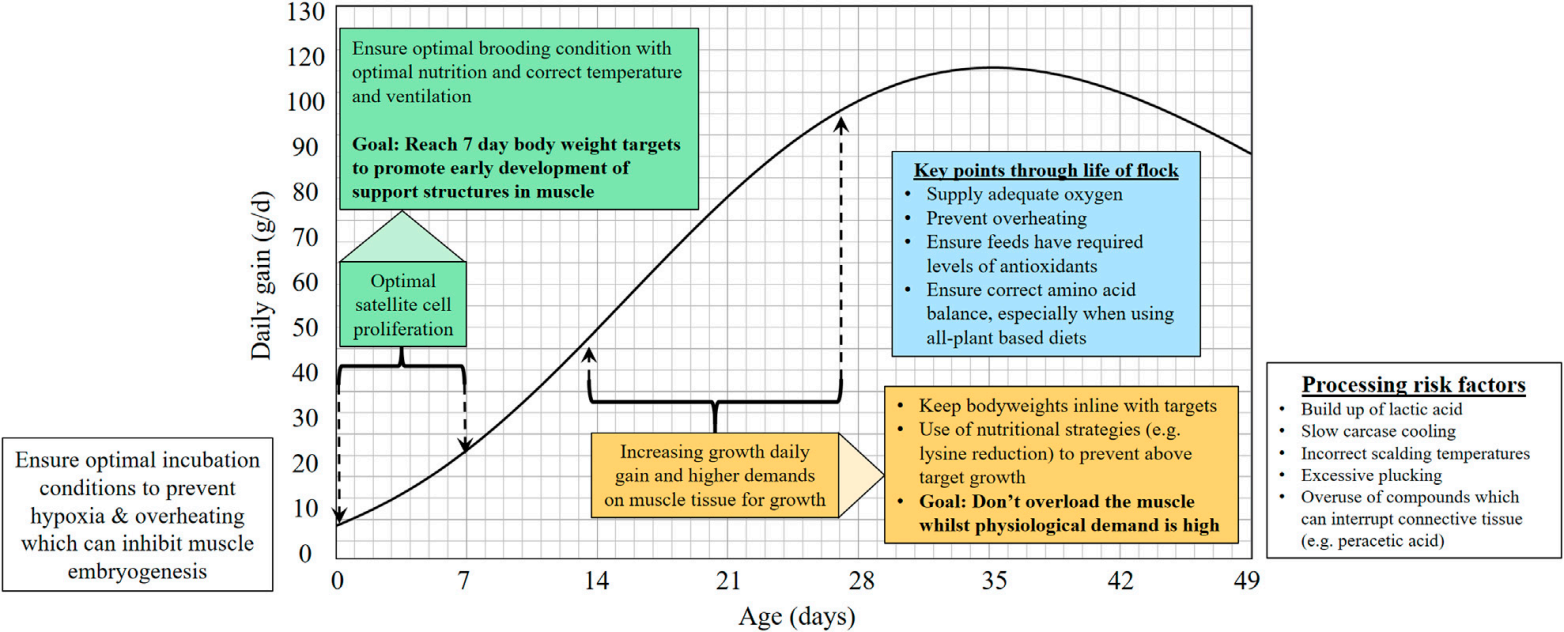
Graph proposing critical stages of broiler lifecycle where management may be critical for reducing myopathies.

Breast myopathies remain an important focus for the poultry industry and the poultry science community, and it is clear that there is still a lot to understand. The reduction in breast myopathies relies on a holistic approach to control: Balanced breeding by poultry breeders can target the genetic component but the larger influence from non-genetic factors remains an important focus area. Understanding the biological needs of the muscle and ongoing physiology in the modern broiler provides key time-points for strategies to reduce the myopathies and gain more insight into their aetiology (Figure 1).
